# On the Influence of Hypodermic Injection upon the Science of Toxicology

**Published:** 1867-06

**Authors:** S. P. Duffield


### II.—ON THE INFLUENCE OF HYPODERMIC INJECTION UPON THE SCIENCE OF TOXICOLOGY.

By S. P. DUFFIELD, Ph. D.

So wide has been the beneficial influence of improvements in chemical analysis, that it would be superfluous to attempt to make any further observations on the important part performed by this branch of the science.

When medicine, in the earlier times, stepped forth and claimed preeminence and respect, while the untiring alchemest, with his *furore*, furnaces and fumes, sought for that elixir which should place eternity within his control, she was then encircled by the *fancies* of her speculative philosophy, encumbering the studies of all her collateral branches.

But in the present age, the exact methods of investigation have plunged her again into a new labyrinth of untenable theories. The famous trial of Palmer, in England, for poisoning his victim with strychnia, drew all chemists to investigate most thoroughly the behaviour of this alkaloid to chemical reagents, and we now have full data and methods which render its detection and recognition quite easy and simple.

The beautiful system of dialysis by Graham has been another step in advance, and can truly be called one of the æsthetics of toxicology, divesting it, as it does, of the curcuitous and very unpleasant course heretofore pursued in examining the viscera of a poisoned subject. But brilliant and rapid as have been the advances in this department of Chemistry, those very discoveries have turned up new obstacles to be overcome.

Of late years, a system of introducing remedial agents into the circulation, more rapidly than can be done through the agency of the stomach, bids fair to place in the hands of designing persons a power which has never before been posessed by any within or without the profession of medicine. I refer to the system of hypodermic injection, becoming now so deservedly popular with the "regular medical profession."

This system owes its success to the facility with which poisons are introduced into the blood.

From the earliest times, *blood* has been a favorite topic.

Moses, in accordance with the views of the ancient Egyptians, placed the seat of life in the blood.

One might, therefore, reasonably have expected that subject which had played such an important part in medicine would have had more than empirical support, on which to base some degree of accurate knowledge.

When we remember that only three-fourths of a century ago oxygen was unknown to the chemist, we can readily perceive why former investigators were powerless. Even to physics, which had solved some of the great astronomical problems, the phenomena of the animal organism were a sealed book.

Albinus took no meagre view of organic activity in nature when he established the axiom, that the essence of life, or the vital force, consisted in *motion*.

Changes are continually going forward in the living body; physical forces are always striving for the equilibrium; the matter set in motion by them finds its centre of gravity—its point of rest. Force is nothing more than the expression of the casual account of natural laws; and if facts do not accord with *our* laws, we have either formed false opinions, or have imperfectly investigated the different circumstances under which they were exhibited.

Within the past few years the science of toxicology, as developed by the German and French chemists, has attained an accuracy which is surprising, when we contemplate the crude state it was in fifty years ago. But rapid as has been its progress, there has suddenly arisen a barrier to its advance, more more formidable than any it had to meet before.

Friedberg and Ritter mournfully acknowledge that the day has not yet arrived when we can detect the difference between dried human and ox blood. A few entuusiasts have claimed a *peculiar oder* to different kinds of blood; but these tests stood on so slender a foundation, and required an almost *hyper-excited* nose to detect them, that no conscientious expert will, for one moment, depend on it for convicting the criminal. The microscope, with its polarizing prism, is not able to distinguish between the most of domesticated animal's blood and that of man, after it has been dried any length of time.

We are not able at the present day to detect *absorbed* alkaloidal poison, and this is the fact forming the subject of this essay, and to which I wish most particularly to call your attention. There can be no doubt that these powerful agents, of which strychnia and morphia are the types, are absorbed into the blood, and diffused throughout the system like other poisons. There seems to be a want of unity in the statements relative to their deposition in the viscera, and their subsequent elimination. M. Stats, in 1847, announced the discovery of the alkaloid in the tissues; but it is questionable whether this was not some portion of the nicotine which had beeu imbibed rather than absorbed. Referring to his process, with which all analytical chemists are familiar, he says he has separated strychnia and brucia from nux vomica, veratria from extract of veratrum, emetina from extract of ipecac, colchicin from wine colchioum, hyoscoamia from extract of henbane, and atropia from extract of belladonia. Some of the poisons mentioned here will destroy life—the fraction of a grain. Mr. Morson, of England, prepares aconitine, of which 1-144 of one grain is the full dose, and says that perhaps the 1-50 would prove fatal to an adult. Where is the analytical chemist who could separate, in quantity enough to give reliable colour test, and obtain crystals, visible even with the strongest microscope, thus 1-50 portion of a grain after it has been thoroughly transfused through twenty-eight pounds of blood, and all the tissues and organs of the body?* He who has this power can detect, and separate, and weigh the specific poison of rabies or of the rattlesnake, and could justly be classed as a rival of Omnipotence itself.

Among numerous cases of poisoning by opium or its alkaloids which have fallen to my lot to examine and depose on, I cannot conscientiously say that I ever detected *absorbed morphia.**

The same remark will apply equally to strychnia, and I cannot see how some men will state deffinitely they can separate *absorbed strychnia*, but have never dared to undertake the task; knowing that the patient dies from the poison absorbed, they, while claiming they can detect and separate *absorbed poison*, contented themselves with extracting the contents of the stomach. When we look back, we find that up to May, 1856, as regarded the detection and separation of strychnia, *chemical science was a blank.* In no once instance before this had strychnia been obtained from the tissues of the corpse, and in the greater number of cases, it had not even been found in its unabsorbed state in the stomach.

With respect, therefore, to the separation of the vegetable poisons, from the blood and tissues, the results are very unsatisfactory. We look in vain in the treatises of Orfila, Kopp, Christison, and in the more recent works of Gauttier, Flandin, Casper, Otto of Braunschweig, Rocker, for any instance in which they claim absorbed strychnia to have been detected either in the human being or in animals; and, in this particular, strychnia is but a type, for the same remarks hold good of other alkaloids.

Dr. Harly, of University College, examined the blood of a dog killed by the 1-12 of a grain of acetate of strychnia injected into the jugular vein. The blood, after the death of the dog, gave no evidence of strychnia. Mr. Hoasly, of Cheltenham, examined the blood and tissues of a dog which he poisoned with two grains of strychnia, and could not detect its presence. Dr. De Vry, of Rotterdam poisoned a dog with nitrate of strychnia, introduced into a wound, and after its death, he examined four ounces of blood, but not the least trace of strychnia was detected. In another case, in which a dog was poisoned in four days by half a grain of strychnia in divided doses, the chemical analysis led to a negative conclusion, not only in the blood and tissues *but in all* parts of the body. Dr. Crawcour, of New Orleans, gave a rabbit half a grain of strychnia; the animal died in half an hour. No trace of the poison was found in any part of the body. In a case of poisoning which occured to Dr. Geoghegan, of Dublin, in 1856, thirty ounces of urine, which had passed the patient from the fifty to the thirty-first hour, when carefully analyzed, did not yield any trace of strychnia. A case of great interest bearing upon this subject occurred to Mr. Wilkins, of Newport, in the Isle of Wright, in February, 1857. A gentleman died in six hours after taking about three grains of strychnia for the purpose of self-destruction. The long period he survived was most favorable for the diffusion and deposition of the poison. The blood and heart were examined by Dr. Taylor and Mr. Scanlan, portions of the liver and lungs were examined by Dr. Christison and Dr. Douglas Maclagan, of Edenburgh, and one kidney was examined by Dr. Geoghegan, of Dublin. The result was, no trace of absorbed strychnia was detected in any one part. In reference to the detection of other alkaloids in an absorbed state, there is an absence of facts. That they enter the blood by absorption is placed beyond a doubt; but whether, when there, they are partially changed, or deposited unchanged in the organs, has not yet been satisfactorily determined by experiment. Dr. De Vry has made recently experiments on the alkaloids, and arrives at the conclusion that that part of the alkaloid which acts mortally is decomposed in the living body. The examination of a large number of cases in the human subject can alone determine perfectly this most important point in toxicology.

Be that as it may, we are absolutely certain of a failure in attempting to detect the poisonous alkaloid atropia in the blood, if administered by hypodermic injection, as it would not require more than one-half a grain to prove fatal. Analytical chemistry, which has, up to this time, occupied so prominent a position, and been so ably associated with forensic medicine, is now perfectly powerless. She cannot solve this problem. There may come a time when more accurate methods and more delecate reagents may lead us to a satisfactory solution of it. Heretofore she has been the Nemesis which pursued, with outstretched grasping hand, the murderer. That hand has been paralyzed by this bold application of principles of chemical physiology in the treatment of disease. The only means now of detection will lie in the testimony of the physician of the symptoms observed by him, at the bedside of the dying person.*

Synthesis is far ahead of analysis, and we must admit that this is a problem of great importance, and to which the attention of toxicologists should be turned. For the present we must say, as we stand grouping on the confines of mortality, and straining our powers to discover in the broad, measureless eternity some means of controlling the moral effect of this fact, and some law which may lead us to processes of detection, that just now we realize how hopeless the human mind is, how utterly futile has been its attempt to discover a mode of detection. In future many a throbbing heart will suddenly cease, and no eye but God's be able to detect the murderer. For the present too much weight cannot be given to the testimony of medical witnesses at the bedside; if that is not had, and no physician was uear at the time of death, we are cast to drift upon an unexplored and perhaps a shoreless sea.

Medical testimony now become all-important, and chemical testimony wanes in value, for no satisfactory results can be obtained. Juries will now, more than ever, be dependent upon circumstantial evidence.

Mortifying in the extreme as it is to our professional pride, stripped of professional honors in medico-legal investigations, the chemist and toxicologist now, if never before, realize the truth that comes floating to us on the dying breath of La Piace —"What we know is little, and what we are ignorant of is immense."—*Proc. Amer. Pharm- Assoc.*

*Equals the 1-9800000 of a grain, assuming *only* the blood contains it. If diffused through the whole body, allowing 128 pounds for tissues, it would then be reduced to the 1-44800000.

*In the case of a woman who committed suicide at one of our hotels and whose stomach was handed me immediately after death, I was able to separate only two grains, when she had actually taken ten grains. Again, in the case of a homœopathic physician who gave solution of morphia, I detected the 1-8 of a grain in a teaspoonful of the solution, and could only get a colour test for morphia from the stomach.

*There is just reason to hope for good results, from analysis, by the spectroscope. Bunsen discovered, in this way, the new metal Cæsium in matter weighing only 1000th part of a grain. —Ed.

